# Prevention of Traumatic Brain Injury in the United States: Significance, New Findings, and Practical Applications

**DOI:** 10.7759/cureus.11225

**Published:** 2020-10-28

**Authors:** Tolulope A Fatuki, Valeriy Zvonarev, Aaron W Rodas

**Affiliations:** 1 Psychiatry/Medicine, Spartan Health Sciences University, Vieux Fort, LCA; 2 Psychiatry, University of Missouri-Kansas City, Hospital Hills Campus, Kansas City, USA; 3 Internal Medicine, Lower Bucks Hospital, Bristol, USA

**Keywords:** tbi, trauma, brain injury, prevention, public health, mva

## Abstract

Traumatic brain injury (TBI) prevention programs aim to reduce trauma-related head injuries across the United States. In addition to epidemiological challenges, patients with TBI have a greater burden of disease and worse health outcomes than the general population. In these circumstances, the prevention of TBI is an important element in reducing the occurrence of post-traumatic health consequences in all settings and beyond. We completed a high-quality overview of TBI prevention programs using the public health approach to identify the most compelling risks to individuals through surveillance, data analysis, and field assessment. We explored the evidence-based programs that are proven to help individuals reduce the risk of TBI. To date, TBI programs have been very efficient, as evidenced by a sustained downturn in TBI incidence. However, recent socioeconomic and epidemiological challenges in the United States are affecting state and local TBI prevention efforts. This article is focused on strategies and solutions to reduce risks and/or consequences associated with head injuries from motor vehicle accidents in New York City. We believe this report is essential to guide the design and implementation of adequate preventive strategies and providing safe and high-quality patient care across all settings where healthcare is delivered.

## Introduction and background

Introduction

Traumatic brain injury (TBI) is the most common type of neurosurgical pathology and is the leading cause of morbidity and disability in adults up to 45 years old. According to some estimates, TBI costs the global economy approximately US $400 billion per year [1]. Currently, there is a pronounced trend of increasing incidence of TBI, which primarily is due to man-made factors’ increased impact on human beings, an increase in criminal injuries, and natural disasters [2].

TBI entails damage to the brain and other intracranial formations (cerebral membranes, blood vessels, cranial nerves) from mechanical force against the head. According to statistics, secondary injuries were recorded in about 90% of patients who died from brain injury. However, even in cases of severe TBI, secondary brain damage can be prevented with timely and adequate therapy [3].

It should be noted that mild TBIs are also a serious medical and social problem, despite the fact that they are characterized by unpronounced neurological symptoms and a relatively short rehabilitation period. Estimated annual incidence of light TBIs worldwide is 100 to 300 people per 100,000 people. Furthermore, significant statistical discrepancies are due to the specificity of diagnostic criteria in different countries [4]. In particular, the data on the incidence with which patients seek treatment for TBIs varies from 0.3% to 44%, and the average length of hospital stays for TBIs varies from three days (Norway) to 21 days (Malta) [5].

Due to the lack of uniform approaches to the classification and diagnosis of TBIs, many cases of light TBIs lack professional attention, which significantly increases the risk of developing chronic neurological and psycho-physiological consequences from trauma. However, numerous studies have shown that the earliest possible medical intervention after a mild TBI significantly reduces the likelihood of delayed chronic effects [5].

All the above make it important to prevent TBIs, as well as improve methods of treating TBIs of differing severity. In this regard, this article aims to compare classical and modern approaches to the prevention of TBIs. As the main criteria for the efficacy of prevention programs, we primarily assessed their scope, the need for financial investments, and changes in targets as a result of program implementation.

Specifically, this type of analysis that we conducted serves to offer partial insight into the actual extent to which traumatic brain injuries have become a threat to the general public in New York City. 

The following objectives have been set to achieve this goal:

- summarize epidemiologic data on TBIs,

- describe the main measures aimed at preventing TBIs,

- consider innovative TBI prevention and prophylactic programs,

- conduct a comparative analysis of several TBI prevention programs’ efficacy,

- describe and analyze the role of motor vehicle accidents and/or dangerous driving behavior in TBI incidence across New York City (NYC),

- attempt to analyze the COVID-19 pandemic’s impact on driving behavior and statistics in NYC, and

- assess these changes’ influence on TBI case.

TBI statistics and epidemiology

TBIs account for 30% to 50% of total traumatic mortality rates. The total mortality rate caused by TBIs, including light and moderate TBIs, is 5% to 10%. Most TBIs occur among people ages 15 to 24 and those over 75 [6,7]. In the United States, the incidence of TBIs varies from region to region and from one social group to another, with a range of 108 to 295 cases per 100,000 people. The highest incidence of TBIs was recorded among men and young people ages 15 to 24 years old, as well as in the older age group [8].

Basic measures for TBI prevention

Primary prevention of TBIs aims to avoid the injury itself, while secondary prevention aims to mitigate the severity of consequences in case of injury. 

Primary prevention programs include interventions that improve vehicle and road infrastructure safety, as well as workplace safety, while secondary prevention programs are based primarily on improving the trauma care system. A practical way to improve primary prevention programs’ efficacy is to target certain risk groups (e.g., programs targeting drivers and cyclists, single elderly people, and children in socially disadvantaged situations) [9,10]. Statutory restrictions on speed limits for road users, improvements in road infrastructure (e.g., separation of pedestrians and cyclists from motorized vehicles), and improved street lighting have been recognized as effective national strategies for TBI prevention [11].

Secondary non-medical prevention strategies include the use of protective headgear and protective systems in vehicles. Mandatory use of a helmet significantly reduces the number and severity of head injuries for both motorcyclists and bicyclists. For example, in Taiwan, the adoption of the Motorcycle Helmet Law in 1997 reduced the number and severity of motorcycle riders’ TBIs by 33%. Educational programs aimed at disseminating information on both preventing TBIs and patient care procedures after TBIs also make an important contribution to preventing TBIs [1].

The main medical measures in secondary prevention of TBIs are based on notions of pathogenetic mechanisms of primary and secondary brain damage caused by mechanical impact. Modern neuroimaging techniques also currently are being used to diagnose TBIs, but in patients with mild TBIs, in approximately 10% of cases, computed tomography may fail to detect morphological signs of brain injury [12]. MRI scans are a more sensitive method of visualizing structural changes, especially if they are performed shortly after TBIs [3].

In accordance with modern ideas about the pathogenesis of TBIs and their complications, processes such as inflammation, receptor-mediated damage, oxidative stress, and brain damage mediated by calcium ions make a major contribution to the destructive processes that occur in brain tissue [13].

It should be noted that the quality of care that a patient receives during the early stages of TBI can be viewed as the primary contributor to patients’ recovery, as well as to reductions in patients’ lifetime healthcare costs [14]. In particular, among TBI patients who were not hospitalized for treatment, approximately twice as many individuals reported restrictions on activity and low life satisfaction compared with patients hospitalized for TBI treatment [15].

After discharge from the hospital, medical and social support aimed at preventing eventual delayed negative consequences comes to the fore [16,17]. These interventions include medication therapy and rehabilitation [4]. The use of alcohol and psychoactive substances is absolutely contraindicated for patients after TBIs, as they can exacerbate existing neurological and psycho-physiological consequences from TBIs significantly [18]. 

TBI prevention and prophylaxis programs

One of the applications of the most inclusive programs implemented at the national level is related to the monitoring of epidemiological indicators of TBIs and identification of their main risk factors. In 1996, the U.S. Congress passed Public Law 104-166, which instructed the federal Centers for Disease Control and Prevention (CDC) to implement TBI prevention projects, which included developing a unified reporting system. This and other tasks related to TBI prevention have received an annual budget of US $3 million between 1997 and 1999, some of which was spent to develop a unified system for collecting information on the incidence and prevalence of TBIs, as well as on TBI-related disabilities in the United States. Until the late 1990s, TBI monitoring systems were implemented in 15 states (Colorado and South Carolina joined later). The integration of data collected from different states based on common clinical criteria has resulted in standardized multi-level TBI assessments. In 2005, TBI surveillance was expanded with additional funding from the Violence and Trauma Prevention Program (Core VTPP), bringing the number of states participating in the program to 30. In addition, 11 states provided the necessary data voluntarily. At the same time, the list of collected data was expanded. 

This system’s implementation has enabled the CDC to identify the most frequent concomitant diseases (e.g., hypertension) and concomitant injuries among those ages 65 and over who have suffered TBIs from falls. Between the late 1990s and mid-2000s, some states used this system to conduct studies on TBIs’ long-term effects [19]. Currently, the TBI program is funded in 20 states, and its data are used widely to analyze different aspects of TBI and confirm that TBI indicators can vary greatly from region to region, even within one country. This needs to be taken into account when analyzing the main risk factors for TBIs. The contribution of different factors to TBI statistics also can vary significantly, which prevents the mechanical transfer of epidemiological data accumulated in one country to other countries, especially if they demonstrate different levels of economic development [20]. Accordingly, the development of national systems for monitoring TBI epidemiology is likely to be viewed as one of the most important modalities of TBI prevention.

Although experts have deemed the algorithm that the CDC developed to be effective, some studies in recent years have demonstrated that its “resolution capacity” can be expanded [21]. In particular, this is evidenced by the comparison of the TBI epidemiological data obtained using the CDC algorithm and the Rochester Epidemiology Project algorithm. The authors estimated the annual TBI incidence per 100,000 people for the years 1987-2000 in Olmstead County, Minnesota. The TBI incidence calculated based on the Rochester Epidemiology Project algorithm and the analysis of medical records was 558 (528 to 590, for a 95% probability level), while the one calculated based on the CDC algorithm was only 341 (331 to 350, for a 95% probability level). However, only 40% of cases with TBI symptoms have been recorded in the CDC system [22].

A special place among TBI prevention programs is occupied by those that aim to improve safety among road users. For example, a significant reduction in the mortality rate from TBIs caused by car accidents has been recorded in several countries among people ages 15 to 24 after implementing phased licensing of new drivers [23].

The CDC has developed a project aimed at selecting the most promising areas for reducing TBIs caused by accidents. The project is based on an interactive online Priority of Intervention and Cost Calculator for States (PICCS). The calculator allows you to select the most effective measures to prevent TBIs caused by accidents from a package of 14 effective tools (seat belt laws, bicycle helmets laws, etc.). PICCS allows for calculating the expected number of injuries and lives saved at the national level, the economic benefits from increasing the number of injuries and lives saved, and the costs of implementing specific measures [19]. The tool is offered publicly to support state and local communities in making evidence-based resource-allocation decisions to help prevent motor-vehicle-related injuries. The tool offers 14 motor vehicle strategies to help reduce motor vehicle injuries. Of the 14 motor vehicle strategies that the tool offers, the State of New York currently uses eight of these 14 motor-vehicle-injury prevention strategies. The six that are not currently in use are “Increased Seat Belt Fines,” “License Plate Impoundment,” Seat Belt Enforcement Campaign,” “In-Person License Renewal,” “Saturation Patrols,” and “Vehicle Impoundment.” Thus, there is still room for improvement in the State of New York to reduce the cost burden from motor vehicle injuries. For example, the “Increased Seat Belt Fines” and “Seat Belt Enforcement Campaign” can be implemented to increase the use of seat belts. In 2014, the DMV’s Statewide Statistical Summary noted that 2.7% of individuals did not use seat belts, totaling 16,068 individuals. Furthermore, the data regarding 57,085 individuals remain unknown. It is important to increase the number of people using seat belts because New York State road statistics in 2014 noted that unrestrained vehicle occupants were three times more likely to be diagnosed with TBIs. The statistics also noted that alcohol-related crashes were associated with the highest risk of TBI. The State of New York already has sobriety checkpoints implemented, but they also can implement saturation patrols to reduce alcohol-related vehicle crashes further [24,25]. 

From our perspective, the most notable results have been achieved through the implementation of programs aimed at improving motorcyclists and bicyclists’ safety, including through the enforcement of mandatory helmet laws. Such programs are most effective in countries with extensive use of motorcycles and bikes, e.g., in Southeast Asia. For example, a comprehensive helmet policy launched in Vietnam in 2007 prevented some 2,200 deaths and 29,000 head injuries, saving $18 million in direct emergency medical care costs. The national program was based on the adoption in 2007 of a package of laws requiring that all riders wear helmets. These laws have expanded the scope of mandatory use of helmets for all road users (not only motorcycle drivers, but also their passengers), significantly increased penalties for refusing to wear a helmet, and provided for several enforcement measures. Fines for failure to wear a helmet have increased from US $2 to $5 to US $11 to $22 (i.e., to about 30% of average monthly per capita income). The average market price for a standard helmet is around $17. As a result of application of these measures, helmet use rose from 30% to 93% in the first few months, and motorcycle-related deaths fell from 29% to 14% [26]. 

Protective helmets’ positive role also has been corroborated through other authors’ findings. Cassidy co-authored research that analyzed two original studies that had been conducted in Australia and in the United States (Seattle, Washington) between 1986 and 1992 [27]. Analysis of the statistics in both cases confirmed that cyclists who used protective helmets were significantly less likely to suffer from TBIs. In Canada, hospital admissions to pediatric wards for children and adolescents with head injuries have decreased by 45% since the enactment of mandatory helmet laws for cyclists. Two other studies that Cassidy analyzed confirmed helmets’ efficacy in preventing TBIs. A meta-analysis of the data published in the early 2000s also confirmed this conclusion [27]. 

Another modality of TBI prevention is prophylaxis programs related to sports and recreation. This modality is enhanced by the fact that the highest incidence of TBIs occurs among youngsters and adolescents [28].

To improve the situation, the CDC has developed and implemented several programs aimed at informing the population about TBIs and changing public attitudes toward TBIs, an example of which is the well-known HeadsUp program [19]. 

HeadsUp targets young athletes, parents, health workers, coaches, and teachers to prevent, recognize, and respond adequately in the event of a TBI. HeadsUp focuses on issues such as TBI prevention and first aid in case of a suspected TBI. The program is based on the transformation of the latest scientific achievements in neurology and education into special educational products specifically designed for the target audience. To date, the CDC has created over 50 such educational products, distributed over 6 million copies of HeadsUp commercials, and prepared over 3 million trainers. Positive changes in high school students’ knowledge levels and behavioral skills related to preventing TBIs have been confirmed [29].

The HeadsUp initiative prepared a tool kit, “Brain Injury In Your Practice,” for health care professionals, but has expanded to provide educational resources that focus on sports programs and schools to reduce the risk of brain injuries, especially in children and teens. One study observed the reach of HeadsUp online material and courses and assessed participants’ knowledge from May 2010 to July 2013. An interesting finding was that New York was one of the states with the lowest participation rates [30]. This does not indicate that coaches in the State of New York are not well-versed on the topic because they may be utilizing different materials to learn and handle concussion management. However, when examining the State of New York, in December 2019, “Public Health Law: Section 2595 Football programs; information on concussions” was passed. It requires all tackle-football programs in the State of New York to provide resources and information packs about brain injuries (concussions and sub-concussive blows) to all parents of children who are participating in tackle-football programs. The State of New York also launched its “When in Doubt…Take Them Out!” prevention campaign, which provides professionals (e.g., coaches, athletes, school administrators) and parents with information to help prevent, recognize, and manage concussions.

The CDC has developed the STEADI program to prevent TBIs in senior citizens caused by falls. STEADI focuses on health professionals working with older people and aims to identify and address modifiable risk factors. By CDC estimates, if 5,000 STEADI-compliant healthcare workers were to examine six million patients over a five-year period, one million falls could be prevented, and $3.5 billion in direct healthcare costs could be saved [31]. STEADI was one of the initiatives implemented in the State of New York in 2012 that focused on identifying patients’ fall risks, assessing any modifiable fall-risk factors, and intervening to reduce fall risks with community and clinically based strategies [32]. In 2014, there were reportedly 29 million falls, and by 2030, one in five Americans will be at least 65 years old. Elderly Americans are more prone to falling, but they are also more susceptible to fall-related injuries, the most common cause of nonfatal trauma-related hospital admissions among older adults. Chronic medical conditions such as cognitive and/or visual impairment, age-associated changes in strength and balance, polypharmacy are also associated with increased risk of falls [32].

In New York, STEADI was initiated in 17 primary care clinics, which soon demonstrated that roughly 70% of older patients were being screened for falls after implementation [32]. Furthermore, another article demonstrated that STEADI can be implemented easily with proper guidance, utilizing the Kotter Framework, an eight-step process for integrating health care initiatives within organizations [33]. The article noted that the focus for a successful initiative depended on aligning a workflow with the usual clinic flow, integrating the STEADI protocol into the EHR, testing the workflow in small sessions before widespread implementation, and implementing in-person training to educate personnel on falls. Once the STEADI protocol was implemented across several teams, within the first three months, they screened 360 patients, i.e., 19% of eligible patients [33]. Prior to this, health care providers had no other fall-screening documents. This study alone offers the opportunity to implement STEADI in all primary care clinics across the State of New York.

Another area of development for prevention programs within the TBI context is the prevention of negative TBI consequences. Such programs are based on identifying factors that increase the risk of maintaining or enhancing symptoms that become more tangible after TBI. One of the preventive programs based on risk factors, the Early Response Brain Injury Service (ERBIS), was implemented at GF Strong Rehabilitation Center in 2003-2004. During the research period, 117 (16.9%) of the TBI individuals included in the program were identified as at risk of prolongation of symptoms and met with the community facilitator for a lecture. During the observation period, these people were divided into two groups: 33 out of 117 people (28%) had permanent post-traumatic symptoms requiring specialized attention, while 84 people (72%) were able to cope with permanent symptoms on their own and gradually returned to their previous routines. Patients who received additional professional help noted an improvement in symptoms after six to 12 months [4].

As the statistics show that TBI is viewed as a factor that increases the risk of suicidal behavior, programs aimed at preventing suicide attempts are of high importance, especially among patients who suffered TBI from military service. Brenner et al. [34] suggested recommendations for developing such programs to be seriously considered by lawmakers.

Modern means of communication currently are used to prevent effects from TBIs. An example of this is a post-discharge TBI patient-support program based on text-message exchanges (SMS). As part of this program, the patient makes a self-assessment of his/her symptoms three times a day in the form of an SMS message, e.g., “9 a.m.: headaches; 1 p.m.: concentration difficulties; 5 p.m.: irritability or anxiety.” In a study of a group of 43 patients who received such support, it was shown that the patients themselves appreciated the importance of this form of support. Compared with the control group, participants in the program had a lower incidence of headaches, attention disorders, irritability, and anxiety. There was also a downward trend in average intensity scores for all the disorders studied, but the differences with the control group were unreliable [35].

## Review

Comparative analysis of TBI prevention programs’ efficacy

Thus far, a large number of TBI prevention programs have been developed and are at different stages of implementation, varying across a whole range of parameters and covering work with the main TBI risk factors. A brief comparative description of the major programs is presented in Table 1.

**Table 1:** Main characteristics of TBI prevention programs Abbreviations: TBI: traumatic brain injury; CDC: Centers for Disease Control and Prevention; PICCS: Priority of Intervention and Cost Calculator for States; STEADI: Stopping Elderly Accidents Deaths and Injuries; ERBIS: the Early Response Brain Injury Service; SMS: short message service.

Program title	Target group	Program objective	Implementation level	Nature of preventive measures
1	2	3	4	5
Programs aimed at reducing TBI incidence				
CDC epidemiological system	20 U.S. states	TBI epidemiology monitoring	Implemented continuously at the federal level	Epidemiological data collection and risk factor identification
Rochester Epidemiological Project	Minnesota	TBI epidemiology monitoring	Implemented continuously at the federal level	Epidemiological data collection and risk factor identification
PICCS	Administrative authorities	Selection of the most effective measures to prevent TBI	Implemented continuously at the federal level	Online calculator for calculating efficiency and economic indicators
HeadsUp	Coaches, athletes, parents	Raising awareness of TBI and prevention measures	Implemented continuously at the federal level	Wide distribution of specially prepared training aids
STEADI	Senior citizens	Creating a safe environment that reduces the risk of falling	Implemented continuously at the federal level	Living arrangements at home, medical support
Programs aimed at reducing the severity of TBIs and their consequences				
Programs related to the use of protective helmets	Motorcyclists, bicyclists	Reduce the likelihood of a heavy TBI in the event of an accident	Implemented in several countries at the federal level	Adoption of laws governing the use of helmets
ERBIS	Patients with TBI	Reduce the risk of development and the severity of complications after the TBI	GF Strong rehabilitation center 2003-2004	GF Strong rehabilitation center 2003-2004
Suicide prevention program	Patients with TBIs caused during military service	Reduce the risk of suicidal behavior	Local, time limited by research time frame	Condition monitoring and psychological support for the patient
Support via SMS	Patients with TBI	Reduce the risk of development and the severity of complications after the TBI	Local	Self-assessment of personal status in the form of responses to automatic SMS messages

We can see that the programs presented in Table 1 significantly differ from each other in their goals, scales, used methods of intervention, etc., which makes the comparison of their efficacy difficult. In addition, basic epidemiological data collection programs are difficult to assess in terms of efficacy because they do not actually impact the target group, but without these programs, it would not be possible to identify groups and risk factors, and, thus, further prevention activities.

In this regard, in Table 2, we present a comparative assessment of the programs reviewed using the criteria that we consider the most relevant.

**Table 2:** Comparative description of prevention programs’ impact on TBI statistics Abbreviations: TBI: traumatic brain injury; CDC: Centers for Disease Control and Prevention; PICCS: Priority of Intervention and Cost Calculator for States; STEADI: Stopping Elderly Accidents Deaths and Injuries; ERBIS: the Early Response Brain Injury Service; SMS: short message service.

Program title	Population outreach	Financial costs	Effect on targets
CDC epidemiological system	+++	+++	Does not involve direct influence
Rochester Epidemiological Project	++	++	Does not involve direct influence
PICCS	++	++	Does not involve direct influence
HeadsUp	++	++	++
STEADI	++	++	++
Programs related to the use of protective helmets	+++	+	+++
ERBIS	+	+	+
Suicide prevention program	+	+	No data
Support via SMS	++	+	++ (according to subjective perceptions of patients)

Based on the data in Table 2, as well as on the data from the analyzed publications, it is possible to consider the programs related to the use of protective helmets to be the most effective among the primary prevention programs. Indeed, these programs do not require significant financial investment (on the contrary, violators will replenish local budgets by paying fines for violations of the law) and cover the entire population of the country or state. Statistics from different countries provide strong evidence of such programs’ efficacy.

From our perspective, programs like HeadsUp and STEADI are highly efficient. From the perspective of the possibility of distributing such programs in rural areas, STEADI can be assumed to be more cost-effective, as it would require less funding because older people are overwhelmingly under the supervision of a district doctor, relatives or social workers. Training doctors and social workers under this program may be sufficient during the first stages of its implementation, but the HeadsUp program envisages substantial investments in the preparation of special training materials.

Among secondary prevention programs, the most promising is the support program through SMS messages. This technology is currently available, relatively inexpensive, and can reach a significant number of patients. Simultaneously, the patients themselves greatly appreciate such a support program. There is no doubt that this program’s efficacy needs to be confirmed, but even with only subjective improvements in patients’ conditions, it can play an important role in their rehabilitation process.

Improvements in dangerous driving behavior as a new mode of TBI prevention

Motor vehicle crashes are the most common cause of emergency room visits, hospitalizations, and deaths related to traumatic brain injury among people ages 15 to 34, according to a 2013 CDC report. Furthermore, more than 14% of cases that the CDC reports are the direct result of automobile accidents. Non-fatal TBIs are the leading cause of hospitalization among those ages 15 to 44 [36].

Reckless driving, that is, using a car in such a way that it “unreasonably interferes with the free and proper use of the public highway” or endangers others, is illegal under New York State law. Additionally, road rage, as defined by the AAA Foundation for Traffic Safety, is still very common among New York drivers [37]. Any unsafe driving performed intentionally and with disregard for safety - such as cutting people off, hitting another car, or physically or verbally assaulting other road users - potentially may lead to traumatic head injuries. Unlike sports or military aspects, little research has been done on the link between car crashes and TBIs. 

This part of our research is designed to assess the hypothesis that aggressive driving behavior among NYC drivers may affect the prevalence of traumas and head injuries. The present analysis aimed to: (1) assess driving behavior characteristics in NYC drivers without cognitive impairments and identify relationships between TBIs and/or traumatic outcomes and on-road behavior, and (2) investigate whether traffic and driving restrictions may improve NYC drivers’ behavior. We attempted to determine the efficacy of the “pause” in predicting the likelihood and frequency of TBI cases during the pre-COVID-19 and lockdown periods. We assumed that tracking data for behavioral trends could improve public safety and add another layer of insight in identifying important aspects needed for improvement. 

It has been suggested that COVID-19-related traffic restrictions affected the incidence of TBIs nationwide, but this has not been proven. Using various public resources, we attempted to predict the COVID-19 lockdown’s impact on driving behavior and TBI cases in New York City. The incidence of car accidents and/or traumatic outcomes in this study has been characterized by this population in terms of borough, time, and crash-related factors. In addition, some characteristics most predictive of TBI during the pre-quarantine and lockdown periods have been identified and compared.

COVID-19 restrictions’ estimated impact on driving behavior and TBI incidence

Most extant studies on the incidence of TBI are inconclusive, and none specifically has discussed brain injuries attributable to motor vehicle crashes and traffic changes during the past three months. Interestingly, a recent study conducted at NYU found mappable correlations between six months of driver behavior data and the numbers related to car crashes. There was a 71% overlap between the location of reckless driving behaviors and motor vehicle accidents around New York City. Moreover, researchers confirmed a 68% correlation between fast acceleration and aggressive driving/road rage collisions, along with hard braking and following-too-closely collisions [38, 39]. 

COVID-19 lockdown restrictions implemented nationwide affected car traffic in many ways. For example, the location technology company TomTom released a traffic index with data from hundreds of U.S. cities showing how these restrictions affected New York City life. It is clear that traffic levels fell sharply in the final weeks of March-April 2020. Unfortunately, the number of deadly automobile crashes in the United States rose dramatically in March-April 2020, even though the number of miles driven decreased due to lockdown time-based traffic restriction (TBR). The National Safety Council concluded that the number of fatal crashes per 100 million miles driven rose an “alarming” 14% compared with March 2019, but the number of traffic deaths in the United States fell by 8% in March compared with 2019 due to an 18.6% drop in miles traveled. While the number of fatal outcomes declined in March 2020, they were still up about 2% for the first three months of 2020. Moreover, several states reported significant increases in speeding. For example, in Los Angeles, speeds were up by as much as 30% on some streets, while authorities in other major metropolitan areas reported drivers traveling at over 100 mph (Tables 8-10 in Appendices). 

Other data provided by Streetlight (www.streetlightdata.com) show significant changes in driving behavior. New Yorkers traveled up to 120.3 million vehicle miles in all five boroughs on March 2. These numbers are very typical for this time of year and characterize the workday before COVID-19. Interestingly, by March 23, the first weekday of the “pause,” the number fell to 26.89 million vehicle miles travelled (VMT), or nearly 78% less. Moreover, total VMTs had dropped to 21.63 million one week later. In contrast, VMTs totaled 29.24 million on May 1, and total VMTs jumped again - by 17% - on May 8. These numbers are significantly higher than the data obtained at the end of March even though nothing had changed in the state’s lockdown restrictions [40,41]. Estimated vehicle miles traveled were down between 78% and 92% compared with January 2020 [42].

In accordance with New York City Mayor Bill de Blasio’s mandates, the city closed several streets to car traffic, expanded sidewalks, and arranged additional bike lanes to provide New Yorkers with extra space to maintain social distancing. New York City Council members also passed a bill to devote approximately 75 miles of city streets to pedestrians throughout the closing. However, this proposal’s safety aspect is still questionable, given the city’s population density [43].

Most people appear to self-monitor their driving behavior, especially during challenging circumstances, such as the pandemic. However, not all drivers can control their driving behavior. According to Anstey et al., factors such as cognition, vision, and other physical functions determine an individual’s driving capacity, but it’s impossible to assess these characteristics fully [44]. However, according to the data provided by Streetsblog NYC, the number of speeding tickets captured by city cameras in residential areas and school zones went up by 57% during the first 10 weekdays after New York started the “pause” [45]. In addition, six cyclists and drivers died in NYC between March 2 and April 9, 2020, the highest number of deaths for the time period since the city’s Vision Zero initiative started five years ago [42]. Other sources reported as much as a 200 percent rise in speeding since the lockdown began [46]. 

Our dataset contained information from all police-reported motor vehicle collisions in NYC during the following two time periods:

(1) January 15 to March 15, 2020, defined as the pre-lockdown period, and

(2) March 16 to May 15, 2020, defined as the lockdown period.

Each row represents a car accident and outlines each event’s characteristics. NYC Open Data Portal (https://opendata.cityofnewyork.us) provided the statistical data on motor vehicle collisions. All numbers and statistical information included in the analysis have been updated and grouped by boroughs. Overall, it is evident that 29,335 car crashes were recorded before the COVID-19 lockdown, which was 75.1 % of the total car crashes, but just 9,719 car crashes were observed during the lockdown period, or only 24.9 % of total car crashes. 

Table 3 and Table 4 represent the number and percentage of injuries in car crashes before and during the COVID-19 lockdown period. During the pre-lockdown period, 17% of car accidents resulted in injuries to one person. Accidents with two injuries totaled 929 (3.2%). Accidents with three injuries totaled 327 (1.1%). Accidents with four injuries totaled 102 (0.3%). Accidents with five or more injuries comprised 0.1% of the total. 78% of crashes during the pre-lockdown period resulted in no injuries.

**Table 3:** Number of injuries during the pre-lockdown period

Number of Injuries	Frequency	Percentage	Valid Percentage	
1	5,009	17.1	78.0	
2	929	3.2	14.5	
3	327	1.1	5.1	
4	102	.3	1.6	
5	34	.1	.5	
6	9	.0	.1	
7	3	.0	.0	
8	5	.0	.1	
9	3	.0	.0	
10	1	.0	.0	
Total	6,422	21.9	100.0	
0	22,913	78.1		

**Table 4:** Number of injuries during the lockdown period

Number of Injuries	Frequency	Percentage	Valid Percentage
1	1,772	18.2	78.8
2	340	3.5	15.1
3	93	1	4.1
4	32	0.3	1.4
5	10	0.1	0.4
7	3	0	0.1
Total	2,250	23.2	100
0	7,469	76.8	

During the lockdown period, accidents with one injury totaled 18%. Accidents with two injuries totaled 340 (3.5%). Accidents with three injuries totaled 93 (1.0%). Accidents with four injuries totaled 32 (0.3%). Accidents with five injuries comprised 0.1% of the total. 77% of car accidents during the COVID-19 lockdown period resulted in no injuries.

**Figure 1:**
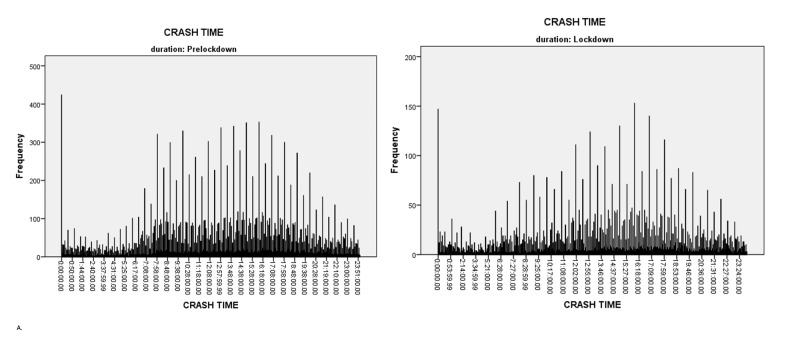
Times of car crashes during the pre-lockdown vs. lockdown period

Figure 1 provides car-crash times during the pre-lockdown period, indicating that crashes peaked between 7:08 and 19:38. Similarly, crashes during the COVID-19 lockdown period peaked between 7:27 and 19:46, as shown in Figure 1. Thus, even though the number of car crashes decreased during the COVID-19 lockdown period, the time of day when they occurred most frequently remained almost the same as before the lockdown.

Table 5 provides an overview of the number of injuries in each borough before and during the COVID-19 lockdown period. As for car accidents in which only one injury was reported, 21% occurred in Brooklyn, 20% in Queens, 12% in the Bronx, 11% in Manhattan and 2% in Staten Island before the lockdown was implemented. The proportions were similar for crashes in which two people were injured, with 22% in Brooklyn and 19% in Queens, followed by the Bronx (9%), Manhattan (6%), and Staten Island (2%). Brooklyn, Queens, and the Bronx remained the deadliest areas, with a higher percentage of car crashes with three, four, and five injuries each. 

**Table 5:** Number of people injured in New York City boroughs during the pre-lockdown period

Number of people injured in New York City boroughs during the pre-lockdown period											
Borough	People Injured										Total
	1	2	3	4	5	6	7	8	9	10	
Not defined	1,735	391	140	53	14	2	3	3	2	1	2,344
	35%	42%	43%	52%	41%	22%	100%	60%	67%	100%	36%
Bronx	594	83	48	10	3	3	0	0	1	0	742
	12%	9%	15%	10%	9%	33%	0.%	0%	33%	0%	11%
Brooklyn	1,055	201	70	14	10	2	0	2	0	0	1,354
	21%	22%	21%	14%	29%	22%	0.%	40%	0%	0%	21%
Manhattan	539	59	10	4	1	0	0	0	0	0	613
	11%	6%	3%	4%	3%	0%	0.%	0%	0%	0%	10%
Queens	982	178	48	16	6	2	0	0	0	0	1,232
	20%	19%	15%	16%	18%	22%	0.%	0%	0%	0%	19%
Staten Island	104	17	11	5	0	0	0	0	0	0	137
	2%	2%	3%	5%	0%	0%	0.%	0%	0%	0%	2%
Total	5,009	929	327	102	34	9	3	5	3	1	6,422
	100%	100%	100%	100%	100%	100%	100%	100%	100%	100%	100%

During the COVID-19 lockdown period, most car crashes with one injury occurred in Brooklyn (23%), followed by Queens (14%) and the Bronx (12%), with very few recorded in Manhattan (7%) and Staten Island (3%). The proportions were similar when two injuries were reported, with 22% in Brooklyn, 17% in Queens, and 8% in the Bronx. Car accidents with three and four injuries were higher in Brooklyn and the Bronx, followed by Queens, Manhattan, and Staten Island. See Table 6 for details. 

**Table 6:** Number of people injured in New York City boroughs during the lockdown period

Borough	Persons Injured										Total
	1	2	3	4	5	6	7	8	9	10	
Not Defined	718	148	48	15	7		2				938
	45%	44%	52%	47%	70%		67%				41%
Bronx	209	28	11	3	1		0				252
	12%	8%	12%	9%	10%		0%				11%
Brooklyn	413	74	19	7	1		1				515
	23%	22%	20%	22%	10%		33%				23%
Manhattan	132	24	4	2	0		0				162
	7%	7%	4%	6%	0%		0%				7%
Queens	255	56	10	5	0		0				326
	14%	17%	11%	16%	0%		0%				15%
Staten island	45	10	1	0	1		0				57
	3%	3%	1%	0%	10%		0%				3%
Total	1,772	340	93	32	10		3				2,250
	100%	100%	100%	100%	100%		100%				100%

Effect on emergency department visits 

According to the National Syndromic Surveillance Program (NSSP), this pandemic affected emergency department (ED) visits nationwide. According to the CDC, ED visits declined 42% during the first month of the pandemic, from a mean of 2.1 million per week (March 31 to April 27, 2019) to 1.2 million (March 29 to April 25, 2020) [47]. These changes have been observed in individuals ≤14 years and female patients. Interestingly, Northeast hospital emergency rooms started seeing a sharp decrease in people coming in with non-COVID-19 issues in early April, a characteristic sign of the public health crisis. Visits to the ER were down 26% in the last week of May compared with a year earlier.

Based on U.S. Department of Health and Human Services data, the largest decline in ED visits was recorded in the Northeast (Region 1, 49%) and in the region that includes New Jersey and New York (Region 2, 48%) [47]. Trauma injuries also were affected during the quarantine, as the number of traffic injuries and outdoor traumas showed a significant decrease during the early stages of the pandemic, then began to increase on February 17. While data on New York and New Jersey remain unavailable, the volume of people entering ERs with various traumas was lower than before the virus.

From March 11 to May 2, 2020, the New York City Department of Health and Mental Hygiene (DOHMH) reported 32,107 deaths, with 24,172 (95% CI = 22,980-25,364) found to be in excess of the seasonal expected baseline. Included in the 24,172 deaths were 13,831 (57%) laboratory-confirmed COVID-19-associated deaths and 5,048 (21%) probable COVID-19-related deaths According to the same data set, 5,293 (22%) excess deaths have not been identified as either laboratory-confirmed or presumed COVID-19-related deaths. It has been concluded that these deaths might have been attributable directly or indirectly to the pandemic and lockdown, but the percentages of deaths from trauma-related impacts from the lockdown remain unknown [48]. 

According to data shared with CNBC, emergency room visits were down by about 50% across New York City Health + Hospital locations. The number of injuries across the city has fallen, as most individuals stayed home under the lockdown order. Moreover, patient visits dropped in the private sector as well, but an unusual uptick in patients who normally seek treatment at hospitals (including various injuries) was recorded [49]. There was also a significant decrease in patients requiring emergency help for conditions like heart attacks and asthma exacerbation. Table 7 provides additional data on the differences in mean weekly numbers of emergency department (ED) visits. 

**Table 7:** Differences in weekly numbers of emergency department (ED) visits for trauma-related diagnostic categories — National Syndromic Surveillance Program, United States, March 31 to April 27, 2019 (comparison period) and March 29 to April 25, 2020 (early pandemic period) [48]

Diagnostic category	Change in mean number of weekly ED visits*	Prevalence ratio (95% CI)
Sprains and strains, initial encounter	−33,709	0.61 (0.61–0.62)
Superficial injuries, contusions, initial encounter	−30,918	0.85 (0.84–0.85)
Other unspecified injuries	−25,974	0.84 (0.83–0.84)

Cumulative results and suggestions

Although road safety has increased greatly over the past years in all NYC boroughs - attributable to improvements in road systems, new laws, and public health and prevention campaigns - enforcement of these laws to improve compliance with suggested regulations has helped reduce the prevalence of car accidents significantly.

Research suggests that young male drivers are most likely to violate basic driving rules and perpetrate road rage. Additionally, the New York State Department of Health reported that young males are almost twice as likely to be hospitalized with a TBI [36]. Although factors such as crowded roads usually boost anger behind the wheel, it has been noted that “stay home” restrictions have not changed this behavior. As previously noted, many professionals suggested that psychological factors such as life stressors may lead to displaced anger [50]. We believe that pandemic-associated stress could lead to similar changes in drivers’ behavior. 

The evidence indicates that when a considerable percentage of cars is restricted from driving, this restriction reduces standard traveling time by up to 30%. Moreover, the policy may shape and improve driving behavior during the regulated period without worsening it during the non-regulated period. The driving restriction should help boost the use of public transit and taxi and/or car-sharing services, but several studies confirmed that such usage would not be statistically insignificant.

To improve traffic conditions further and reduce the number of traumatic outcomes, NYC may impose rules barring cars from the busiest areas based on license plate numbers to control traffic congestion. The government also should set the total number of car licenses that can be issued per year at the city level. We believe that these preventive measures may limit the growing number of car owners in NYC. While all these measures may be effective in theory, we cannot estimate whether this policy would be effective enough in the short run to justify implementation in NYC.

During our analysis, it was noted that most similar extant research focused on crash-causing behaviors instead of everyday driving behaviors. Moreover, no research has focused on driving errors that typical car owners and riders make on a daily basis. This means that we could not fully assess driving behavior due to a lack of any standards. It is imperative to determine what kind of driving “errors” New Yorkers have made and whether these issues have been dangerous or simply “normal habits” that most city drivers make at some point in their lives. It remains unclear how to score different types of behavior or “driving errors.” It makes on-road tests’ reliability questionable, which, in turn, suggests that these tests may have low validity. We believe that future research should focus on the observation and assessment of citizens’ actual driving behavior using on-road assessment strategies. This approach would help distinguish between safe and unsafe driving, which is critical to New Yorkers’ safety.

## Conclusions

Public health initiatives coordinated across the city and communities could help to prevent the occurrence of TBIs. However, current data do not provide enough details needed to fully understand the epidemiology and potential outcomes of TBI prevention programs. Nevertheless, overall decreases in TBI-related hospitalizations and deaths due to MVA indicate significant prog­ress of TBI-prevention efforts. Moreover, it has been suggested that the quarantine and transportation restrictions introduced in New York City decreased the incidence of MVA - related injuries in several areas. To improve traffic conditions and indirectly affect the incidence of TBI cases, we concluded that NYC should implement certain measures such as a driving restriction policy. The rates of TBI-related deaths due to intentional self-harm and/or falls remain high, which reflects the increase in suicide rates across the United States, suggesting the need for expansion of comprehensive and well-coordinated TBI- prevention efforts, but in different directions.

## Appendices

**Table 8:** Motor-Vehicle Deaths and Changes United States, Three Months, 2017 to 2020

March 2020 Motor-Vehicle Deaths and Changes United States, Three Months, 2017 to 2020*										
Month	Number of Deaths				Percent Changes					
	2017	2018	2019	2020	Corresponding Month				Four Month Moving Average +	
					2018 to 2020		2018 to 2019	2019 to 2020	20118 to 2019	2019 to 2020
January	3,034	3,010	2,830	2,900		-4%		2%		<0.5%
February	2,748	2,734	2,590	2,870		5%		11%		4%
March	3,164	3,015	2,910	2,690		-11%		-8%		1%
3 Months	8,946	8,759	8,330	8,460		-3%		2%		
April	3,238	2,979	3,040				2%		-3%	
May	3,416	3,443	3,410				-1%		-2%	
June	3,492	3,514	3,420				-3%		-1%	
July	3,730	3,552	3,530				-1%		-1%	
August	3,409	3,490	3,570				2%		<0.5%	
September	3,572	3,579	3,520				-2%		-1%	
October	3,629	3,657	3,430				-6%		-2%	
November	3,408	3,250	3,340				3%		-1%	
December	3,391	3,181	3,210				1%		-1%	
TOTAL	40,231	39,404	38,800	38,930	#					
NOTE: National Safety Council figures are not comparable to National Highway Traffic Safety Administration figures. NSC counts both traffic and nontraffic deaths that occur within a year of the accident, while NHTSA counts only traffic deaths that occur within 30 days. The 2017 and 2018 data are from the National Center for Health Statistics. All other figures are National Safety Council estimates. *Latest updates: 2017--12/14/18; 2018--2/13/19; 2019--2/17/20. +Four-Month Moving Average is based on changes between the totals of four consecutive months. Adding several months together tends to smooth out single-month changes that may be affected by differences in the number of weekends in a month from one year to the next or by other random variations. #Deaths for the 12-month period ending March 2020										

**Table 9:** State motor-vehicle deaths and percent changes

State motor-vehicle deaths and percent changes						
State	Number of Months Reported	Deaths Identical Periods			Percent Changes	
		2020	2019	2018	2019 to 2020	2018 to 2020
TOTAL U.S.	3	8,460	8,330	8,759	2%	-3%
Alabama	3	197	190	229	4%	-14%
Alaska	3	13	17	13	-24%	0%
Arizona	3	232	241	265	-4%	-12%
Arkansas	3	114	98	87	16%	31%
California	3	685	633	542	8%	26%
Colorado	3	101	95	120	6%	-16%
Connecticut	3	68	48	52	42%	31%
Delaware	3	23	18	21	28%	10%
DC	3	7	3	7	133%	0%
Florida	3	894	882	866	1%	3%
Georgia	3	366	354	326	3%	12%
Hawaii	3	21	31	25	-32%	-16%
Idaho	3	28	39	29	-28%	-3%
Illinois	3	188	170	239	11%	-21%
Indiana	3	146	154	189	-5%	-23%
Iowa	3	54	62	61	-13%	-11%
Kansas	3	96	93	88	3%	9%
Kentucky	3	138	149	152	-7%	-9%
Louisiana	3	170	138	163	23%	4%
Maine	3	28	32	12	-13%	133%
Maryland	3	90	104	89	-13%	1%
Massachusetts	3	69	73	81	-5%	-15%
Michigan	3	174	198	177	-12%	-2%
Minnesota	3	68	67	57	1%	19%
Mississippi	3	125	127	135	-2%	-7%

Missouri	3	175	163	184	7%	-5%
Montana	3	27	20	21	35%	29%
Nebraska	3	41	44	49	-7%	-16%
Nevada	3	66	60	73	10%	-10%
New Hampshire	3	27	14	21	93%	29%
New Jersey	3	123	119	119	3%	3%
New Mexico	3	101	103	77	-2%	31%
New York	3	149	127	148	17%	1%
North Carolina	3	314	285	325	10%	-3%
North Dakota	3	7	17	13	-59%	-46%
Ohio	3	231	240	224	-4%	3%
Oklahoma	3	131	120	146	9%	-10%
Oregon	3	75	99	94	-24%	-20%
Pennsylvania	3	222	233	284	-5%	-22%
Rhode Island	3	12	13	11	-8%	9%
South Carolina	3	186	211	189	-12%	-2%
South Dakota	3	18	13	24	38%	-25%
Tennessee	3	240	227	209	6%	15%
Texas	3	861	813	893	6%	-4%
Utah	3	56	48	54	17%	4%
Vermont	3	6	3	7	100%	-14%
Virginia	3	189	183	182	3%	4%
Washington	3	96	119	100	-19%	-4%
West Virginia	3	56	55	57	2%	-2%
Wisconsin	3	84	99	130	-15%	-35%
Wyoming	3	7	35	20	-80%	-65%
NOTE: Deaths are reported by state traffic authorities. All figures are preliminary. To ensure proper comparisons, 2018 and 2019figures are preliminary figures covering the same reporting period as those for 2020. The total for 2018 is from the National Center for Health Statistics.						

**Table 10:** Preliminary monthly fatality totals reported by states*, 2019-2020

Preliminary monthly fatality totals reported by states*, 2019-2020				
Year	State	January	February	March
2020	Alaska	4	6	3
2020	Alabama	56	70	71
2020	Arkansas	39	36	39
2020	Arizona	76	105	51
2020	California	278	244	163
2020	Colorado	32	35	34
2020	Connecticut	24	23	21
2020	Delaware	10	8	5
2020	Dist. of Columbia	5	2	0
2020	Florida	318	275	301
2020	Georgia	102	126	138
2020	Hawaii	12	5	4
2020	Iowa	24	17	13
2020	Idaho	6	14	8
2020	Illinois	76	65	47
2020	Indiana	47	41	58
2020	Kansas	30	35	31
2020	Kentucky	45	42	51
2020	Louisiana	58	50	62
2020	Massachusetts	21	20	28
2020	Maryland	20	31	39
2020	Maine	16	6	6
2020	Michigan	64	62	48
2020	Minnesota	18	22	28
2020	Missouri	50	73	52
2020	Mississippi	37	51	37
2020	Montana	10	4	13
2020	North Carolina	117	88	109
2020	North Dakota	2	1	4

2020	Nebraska	14	17	10
2020	New Hampshire	10	8	9
2020	New Jersey	46	41	36
2020	New Mexico	41	28	32
2020	Nevada	24	25	17
2020	New York	71	51	27
2020	Ohio	77	69	85
2020	Oklahoma	42	45	44
2020	Oregon	35	22	18
2020	Pennsylvania	90	60	72
2020	Rhode Island	4	3	5
2020	South Carolina	62	65	59
2020	South Dakota	5	10	3
2020	Tennessee	74	89	77
2020	Texas	307	304	250
2020	Utah	15	19	22
2020	Virginia	72	62	55
2020	Vermont	3	1	2
2020	Washington	38	28	30
2020	Wisconsin	25	30	29
2020	West Virginia	19	23	14
2020	Wyoming	2	1	4
2019	Alaska	7	4	6
2019	Alabama	74	43	73
2019	Arkansas	38	25	35
2019	Arizona	84	75	82
2019	California	274	190	169
2019	Colorado	32	24	39
2019	Connecticut	21	11	16
2019	Delaware	5	4	9
2019	Dist. of Columbia	0	2	1

2019	Florida	254	294	334
2019	Georgia	126	96	132
2019	Hawaii	14	10	7
2019	Iowa	21	20	21
2019	Idaho	8	21	10
2019	Illinois	52	39	79
2019	Indiana	42	71	41
2019	Kansas	33	21	39
2019	Kentucky	50	53	46
2019	Louisiana	47	50	41
2019	Massachusetts	21	30	22
2019	Maryland	30	44	30
2019	Maine	15	8	9
2019	Michigan	76	55	67
2019	Minnesota	26	18	23
2019	Missouri	59	52	52
2019	Mississippi	44	45	38
2019	Montana	12	4	4
2019	North Carolina	101	89	95
2019	North Dakota	3	4	10
2019	Nebraska	9	18	17
2019	New Hampshire	6	4	4
2019	New Jersey	43	40	36
2019	New Mexico	32	34	37
2019	Nevada	23	17	20
2019	New York	56	39	32
2019	Ohio	72	69	99
2019	Oklahoma	43	31	46
2019	Oregon	32	29	38
2019	Pennsylvania	79	79	75
2019	Rhode Island	7	3	3

2019	South Carolina	83	59	69
2019	South Dakota	4	3	6
2019	Tennessee	80	82	65
2019	Texas	272	247	294
2019	Utah	15	11	22
2019	Virginia	65	60	58
2019	Vermont	2	0	1
2019	Washington	48	28	43
2019	Wisconsin	37	34	28
2019	West Virginia	19	14	22
2019	Wyoming	14	4	17
*NOTE:	Deaths are reported by state traffic authorities. All figures are preliminary. To ensure proper comparisons, 2019 fatality estimates are preliminary figures covering the same reporting period as those for 2020.			
